# Trends in viral hepatitis liver-related morbidity and mortality in New South Wales, Australia

**DOI:** 10.1016/j.lanwpc.2024.101185

**Published:** 2024-08-31

**Authors:** Shane Tillakeratne, Sallie-Anne Pearson, Maryam Alavi, Behzad Hajarizadeh, Marianne Martinello, Matthew Law, Jacob George, Janaki Amin, Gail Matthews, Jason Grebely, Gregory J. Dore, Heather Valerio

**Affiliations:** a The Kirby Institute, UNSW Sydney, Australia; b Department of Health Sciences, Faculty of Medicine and Health Sciences, Macquarie University, Sydney, NSW, Australia; c School of Population Health, UNSW Sydney, Australia

**Keywords:** DC, HCC, Hepatitis B, Hepatitis C, Mortality, Liver disease, Population-level

## Abstract

**Background:**

Monitoring hepatitis B virus (HBV) and hepatitis C virus (HCV) liver-related morbidity and mortality is key to evaluate progress towards elimination targets.

**Methods:**

HBV and HCV notifications in NSW, Australia (1995–2022) were linked to hospital and mortality records. Temporal trends in decompensated cirrhosis (DC), hepatocellular carcinoma (HCC), and mortality were evaluated among people notified for HBV and HCV. Segmented Poisson regression models were used to assess the impact of the viral hepatitis elimination era (1 January 2015–31 December 2022) on advanced liver disease and mortality.

**Findings:**

During 1995–2022, there were 64,865 people with an HBV notification and 112,277 people with an HCV notification in NSW. Between 2002 and 2022, there were significant reductions in age-adjusted HBV- and HCV-related DC, HCC, and liver-related mortality. Among those with HBV, age-standardised incidence per 1000 person-years (py) in 2002, 2015, and 2022 was 3.08, 1.47, and 1.16 for DC (p < 0.001); 2.97, 1.45, and 0.75 for HCC (p < 0.001); and 2.84, 1.93, and 1.40 for liver-related mortality (p < 0.001). Among those with HCV, age-standardised incidence per 1000 py in 2002, 2015, and 2022, was 5.53, 4.57, and 2.31 for DC (p < 0.001); 2.22, 2.59, and 1.87 for HCC (p < 0.001); and 3.89, 4.73, and 3.16 for liver-related mortality (p < 0.001). In 2022, absolute liver-related mortality per 100,000 population was 0.95 for HBV and 3.56 for HCV. In adjusted analyses, older age, comorbidity, and a history of alcohol use disorder were associated with increased liver-related mortality among those with HBV and HCV.

**Interpretation:**

This population-level study demonstrated declining risks of DC, HCC, and mortality, with HBV-related declines commencing well before elimination era while HCV-related declines were mostly during elimination era. Population liver mortality indicates elimination target achieved for combined viral hepatitis and HBV, but not HCV.

**Funding:**

The Kirby Institute, 10.13039/501100001773UNSW Sydney, and New South Wales Ministry of Health, Australia.

**Research in context**

**Evidence before this study**

We searched Medline, Scopus, Web of Science using key search terms for papers published between January 1995 and March 2024. Previous population-based studies published in the early DAA era for hepatitis C virus (HCV) demonstrated early impact of DAA therapy on HCV-related morbidity and mortality. Few population-based studies have evaluated trends in viral hepatitis-related decompensated cirrhosis, hepatocellular carcinoma, and mortality among people with hepatitis C virus (HBV) or HCV. Furthermore, we found no studies evaluated progress toward viral hepatitis elimination collectively in relation to both the World Health Organization's 2015 call for HBV and HCV elimination by 2030 and the revised mortality targets. This highlighted a significant gap in research crucial for assessing progress toward viral hepatitis elimination objectives.

**Added value of this study**

This study offers critical updates and comprehensive data on viral hepatitis-related liver disease and mortality trends, aligning with the World Health Organization's revised mortality targets. It utilises a robust data linkage system in New South Wales, Australia, integrating all HBV and HCV notifications, and corresponding hospitalisations, and mortality data, providing high precision in tracking viral hepatitis elimination. Our findings demonstrate declines in severe liver disease incidence and mortality rates following the availability of potent antiviral drugs and improved clinical management. This study highlights the differentiated impact on hepatitis B and C populations, underscoring the effectiveness of current interventions while identifying continued areas of need. The continued elevated risk of liver-related mortality among those with alcohol use disorder and multiple comorbidities was also highlighted, emphasising the need for targeted behavioural interventions. Overall, the study suggests progress toward achieving the WHO's revised mortality targets through strategic interventions and enhanced clinical management.

**Implications of all the available evidence**

The WHO has set a goal to eliminate viral hepatitis a major global public health threat by 2030, including a reduction in HBV and HCV mortality rates to ≤4 and ≤ 2 per 100,000 population, respectively. Our study underscores the benefits of early, unrestricted access to antivirals and improved clinical management, highlighting their crucial role in extending treatment reach and effectiveness. High coverage and monitoring of antiviral treatment is essential for averting progression to advanced liver disease. These findings emphasise the need for public health approaches to enhance viral hepatitis elimination on the population scale, as well as targeted interventions for populations with significant comorbidity. This study provides robust data for shaping future research, policy decisions, and practice guidelines to achieve global viral hepatitis elimination targets. Policymakers must consider these insights to allocate resources effectively and implement comprehensive strategies addressing medical and social determinants of health to reduce the viral hepatitis burden and reach elimination by 2030.

## Introduction

The World Health Organisation (WHO) has committed to eliminate viral hepatitis as a public health threat by 2030.^1^^,^^2^ Elimination as defined by WHO, encompasses specific impact targets including reductions in new infections and mortality attributed to hepatitis B virus (HBV) and hepatitis C virus (HCV).^2^^,^^3^

Elimination of viral hepatitis is possible, in part due to enhanced potential of antiviral therapy to avert severe liver-related morbidity and mortality.^4^, ^5^, ^6^ In Australia, more potent HBV antiviral therapy was subsidised from 2007,^7^ and HCV direct-acting antiviral (DAA) regimens in 2016.^8^^,^^9^ Despite evidence of decreased liver morbidity and mortality in Australia,^10^^,^^11^ recent modelling has indicated the need for enhanced interventions to achieve elimination by 2030.^12^, ^13^, ^14^

In 2016 the mortality target was defined, as a relative reduction (65% reduction in deaths attributable to HBV or HCV between 2015 and 2030).^2^ Attaining this reduction was problematic in most settings due to pre-2015 mortality upward trajectory.^14^ Moreover, the quality of baseline data significantly influences the precision with which progress can be monitored.^15^

As such, the recent revision to absolute HBV- and HCV-related mortality elimination targets of ≤4 and ≤ 2 per 100,000 population, respectively,^16^ is welcomed, providing a more universally applicable framework.^14^^,^^15^ There is complexity of monitoring mortality trends, particularly in settings where data, are either lacking or less than optimal.^15^ New South Wales (NSW), Australia, however, is one of few settings globally with person level-linkages to all HBV and HCV notifications. Therefore, the objective of this paper was to evaluate the impact of the WHO's call for viral hepatitis elimination on HBV and HCV-related DC, HCC incidence and mortality, and overall progress towards achieving the revised mortality targets in New South Wales, Australia.

## Methods

### Data sources and linkage

The NSW Notifiable Conditions Information Management System (NCIMS) contains records of individuals with HBV or HCV notification since 1993. A notification represents a confirmed case of hepatitis B virus (HBV) or hepatitis C virus (HCV) infection, as reported by healthcare providers or laboratories in NSW based on positive diagnostic test results. The NSW Admitted Patient Data Collection, covering all inpatient admissions and diagnosis data since 2001. Death registrations in NSW are available through the Registry of Births, Deaths, and Marriages since 1993. Probabilistic record linkage utilising both automated and algorithmic blocking and scoring methods (scoring computed using a machine learning technique, and absolute rules using ChoiceMaker software^17^) were employed by the NSW Centre for Health Record Linkage to link hospitalisation and mortality records for all people with HBV or HCV notification. Scoring methods utilised by ChoiceMaker included upper and lower probability cut-offs, to determine the final decision as to whether each potential match denotes or possibly denotes the same person. Upper and lower probability cut-offs started at 0.8 and 0.45 for a linkage and were adjusted for each individual linkage to ensure minimal false links. Of all records included in the final linkage, a false positive rate of 0.5% was reported.

### Observation period

HBV and HCV notifications were extracted for the study period January 1, 1993, to March 31, 2022. Linked hospitalisation records were extracted for the study period January 1, 2002, to December 31, 2022, while linked mortality records were extracted for the study period January 1, 1993, to December 31, 2022.

### Inclusion criteria

All individuals with an HBV or HCV notification in the NCIMS were included in the study, regardless of age at the time of notification. Individual-level treatment linked data was not available for analysis. Consequently, analyses stratified by treatment for HBV and HCV were not included, and individuals treated and cured are contained within the cohorts.

### Exclusion criteria

To address potential data quality issues and reduced antibody specificity with early assays, HBV and HCV notifications occurring prior to 1995 were removed (n = 19,034). Post-mortem HBV and HCV notifications were excluded (n = 557), as were notifications with death records occurring before the start of hospitalisation data (January 1, 2002) (n = 1793). Records with missing date of birth (n = 794) and sex (n = 103) (<0.005% of all records) were included in the final cohort analyses, however, were excluded from age-standardisation calculations.

### Study outcomes

The outcomes of interest include HBV or HCV-related decompensated cirrhosis (DC, HBV-DC; HCV-DC) and hepatocellular carcinoma (HCC, HBV-HCC; HCV-HCC) diagnoses, and HBV or HCV-related liver and all-cause mortality.

Inpatient hospital records were used to identify DC and HCC diagnoses. Reasons for each hospital episode were recorded using ICD-10 with primary and secondary codes included (up to 50 diagnostic fields). For DC, diagnostic codes included ascites (R18), oesophageal varices with bleeding (I85.0 and I98.3), chronic hepatic failure (K72.1 and K72.9), alcoholic hepatic failure (K70.4), and hepatorenal syndrome (K76.7) (Table S1). The relevant diagnosis code for HCC was liver cell carcinoma (C22.0).^10^

Liver-related mortality was defined using a previously validated method,^10^^,^^18^ where any death following DC or HCC diagnosis is classified as liver-related.^18^ All-cause mortality was defined as any record of death.

### Population demographics and characteristics

Factors of interest included year of birth, birth cohort (≥1967 and < 1967), median age (Diagnosis of DC/HCC, death), person years at risk (PYAR), sex (male, female), history of alcohol-use disorder (AUD),^19^ Charlson co-morbidity index (CCI) (3+),^20^^,^^21^ and the elimination era (1 January 2015–31 December 2022). PYAR were calculated from January 1, 2002 for individuals with HBV/HCV notifications prior to this date, and from the date of notification for subsequent cases, with follow-up continuing until death, diagnosis of DC or HCC, or the end of the follow-up period, 31 December 2022. AUD is a standard term used to define continued drinking despite adverse consequences.^19^ For analyses, having a history of at least 1 AUD-related hospital admission will be referred to as AUD. Codes used to diagnose AUD are presented in Table S2. CCI is a standard tool used to assess and quantify the burden of comorbid conditions in individuals, aiding in the prediction of overall disease severity. The selection of 1 January 2015 as the start of the elimination era was chosen for HBV and HCV to align with the WHO baseline relative elimination targets.

### Statistical analysis

#### Analysis 1: characteristics of people with HBV or HCV notification made in NSW, Australia

The changing demographics and characteristics of the HBV and HCV notified population were assessed according to the following time periods: January 1, 2002–December 31, 2007; January 1, 2008–December 31, 2014; and January 1, 2015–December 31, 2022. These periods were selected to reflect major shifts in treatment landscapes in Australia: 2002–2007 captures the early HCV interferon-combination era and early HBV antiviral therapy (including lamivudine), 2008–2014 captures improved HBV therapies (including tenofovir and entecavir),^8^ while 2015–2022 represents the viral hepatitis elimination era and introduction and subsequent subsidisation of HCV direct-acting antiviral therapy.^22^

#### Analysis 2: temporal trends of absolute decompensated cirrhosis, hepatocellular carcinoma diagnoses, and liver-related and all-cause mortality among people with HBV or HCV notification

Trends in HBV- and HCV-DC and -HCC diagnoses, and liver-related and all-cause mortality were plotted between 2002 and 2022. Liver-related and all-cause mortality was calculated per 100,000 population and compared with the WHO HBV and HCV absolute mortality targets.

#### Analysis 3: age-standardised incidence rates of decompensated cirrhosis, hepatocellular carcinoma diagnoses, liver-related and all-cause mortality among people with HBV or HCV notification

Annual incidence of HBV- and HCV-DC, -HCC, and liver-related and all-cause mortality was calculated. Time at risk for DC, HCC, and liver-related and all-cause mortality started at first recorded HBV or HCV notification date and was censored on first date of DC or HCC diagnosis, date of death or the conclusion of follow-up period (31 December 2022). Incidence rates (per 1000 person-years [PY]) for each outcome were age standardised using Australian standard population, 2013.^23^ Rates are presented with corresponding 95% confidence intervals and were calculated assuming a Poisson distribution.

Given the potential of alcohol to impact liver morbidity and mortality,^24^^,^^25^ a supplementary analysis was performed to stratify its potential contribution to HCV-related liver morbidity (DC and HCC) and mortality. Trends and age-standardised incidence rates (per 1000 PY) of DC and HCC diagnoses were compared among individuals with and without evidence of AUD.

#### Analysis 4: evaluating the impact of the viral hepatitis elimination era among people with HBV or HCV notification

Segmented Poisson regression models, fitting a second time trend parameter using splines, were used to assess the impact of the viral hepatitis elimination era on the number of DC diagnoses and HCC diagnoses, and liver-related and all-cause mortality. The analysis covered the period from 1 January 2002 to 31 December 2022, with the date of HBV or HCV notification serving as the entry date for each case. The change point of interest was set on 1 January 2015, which previously served as the baseline for relative WHO elimination mortality targets. Data was segmented into 6-monthly intervals and categorised as pre-elimination era (1 January 2002–31 December 2014) or elimination era (1 January 2015–31 December 2022). Results are presented with observed data, predicted mean, and predicted mean assuming no intervention (counterfactual), i.e., without the WHO's call for viral hepatitis elimination. Models were adjusted for age group (<20, 20–39, 40–59, 60–79, 80+ years) and sex, with inclusion of person-years at risk as an offset term. The segmented regression model estimated the temporal trends in HBV and HCV-related DC, HCC, liver-related and all-cause mortality in both the pre-elimination and elimination eras, as well as the change in trend between the two periods. Results are presented as count ratios (CRs) with 95% confidence intervals (CIs) and associated p-values. In addition, the cumulative number of HBV and HCV-related cases averted during the elimination era (2015–2022) was estimated by comparing the observed number of cases to the projected number based on pre-elimination trends.

#### Analysis 5: factors associated with liver-related mortality among people with HBV and HCV

Finally, factors associated with liver-related mortality were assessed using unadjusted and adjusted Poisson regression. For analyses comprising the HCV notified population, coinfection with HBV was also assessed.

Statistical analyses were performed using Stata, version 14.2 (College Station, TX, USA) Visualisation of data was presented using GraphPad Prism 10.2.2 (Insight Partners, NY, USA). Smoothing of age-standardised incidence rates was performed utilising the Savitzky–Golay smoothing filter (second-order polynomial, window size [5])^26^ to enhance visual clarity while preserving underlying trends in the data.

### Ethics

This study was approved by NSW Population and Health Services Research Ethics Committee (Approval number: 2019/ETH01777) and the Australian Institute of Health and Welfare Ethics Committee (Approval number: EO2021/3/1274).

### Role of the funding source

The funder of the study had no role in study design, interpretation, writing of the report or the decision to publish this study. The corresponding author had full access to all the data in the study and had final responsibility for the decision to submit for publication.

## Results

During 1995–2022, there were 64,865 people with an HBV notification and 112,277 people with an HCV notification in NSW (Table 1, Table 2). Among those with HBV, median year of birth was 1970 (interquartile range [IQR], 1958–1980), 54% (n = 35,262) were male, 2% (n = 1516) had a history of AUD, and 6% (n = 3776) had a CCI score of 3 or higher (Table 1). Nine percent (n = 3735) died, and among those who died, median age at death was 68 years (interquartile range 56–79) (Table 1). Among those with HCV, median year of birth was 1967 (IQR, 1959–1977), 64% (n = 72,047) were male, 22% (n = 24,100) had a history of AUD, 9% (n = 10,013) had a CCI score of 3 or higher, and 4% (n = 4346) were also notified with HBV. Sixteen percent (n = 16,701) died; among those who died, median age at death was 55 years (IQR, 46–64) (Table 2). Additionally, males had a higher prevalence of AUD (24%; n = 11,926) compared to females (17% n = 6597) (Table S3).

**Table 1:** Demographic characteristics of people with an HBV notification, by type of liver disease and time period.

	all HBV^a^	Period of DC and HCC diagnosis								
		2002–2007			2002–2007			2002–2007		
		all HBV	DC	HCC	all HBV	DC	HCC	all HBV	DC	HCC
Characteristics, n (%)	n = 64,865	n = 14,620	n = 271	n = 221	n = 15,232	n = 327	n = 361	n = 13,241	n = 401	n = 465
Person-years at risk^b^	1,002,304	174,341			473,904			919,021		
Year of birth, median (IQR)^c^^,^^d^	1970 (1958–1980)	1969 (1958–1977)	1949 (1940–1959)	1947 (1937–1955)	1975 (1963–1983)	1952 (1942–1961)	1953 (1944–1961)	1979 (1965–1987)	1956 (1947–1964)	1955 (1948–1962)
Age at diagnosis, median (IQR)^c^^,^^d^^,^^e^	60 (51–69)	59 (49–67)	56 (45–65)	58 (50–67)	59 (50–68)	59 (49–68)	59 (50–67)	62 (54–71)	63 (54–71)	64 (56–71)
Male sex^d^	35,262 (54)	8099 (56)	201 (74)	185 (84)	8202 (54)	255 (78)	305 (85)	7185 (54)	295 (74)	372 (80)
Female sex^d^	29,290 (45)	6455 (44)	69 (25)	35 (15)	6955 (46)	70 (22)	55 (15)	6030 (45)	105 (26)	92 (20)
AUD	1516 (2)	442 (3)	52 (19)	18 (8)	334 (2)	60 (18)	26 (7)	224 (2)	68 (17)	37 (8)
Death	3748 (6)	235 (7)	172 (63)	129 (58)	275 (2)	209 (64)	177 (49)	396 (3)	293 (73)	210 (45)
Age at death, median (IQR)^c^^,^^d^	68 (56–79)	65 (54–78)	59 (50–68)	61 (52–71)	67 (55–77)	62 (53–73)	62 (53–73)	69 (58–79)	66 (56–73)	64 (57–72)
Charlson comorbidity index 3+^f^	3776 (6)	877 (6)	114 (42)	78 (35)	763 (5)	154 (47)	137 (38)	565 (4)	210 (52)	138 (30)

Demographic characteristics of people with an HBV notification, by type of liver disease and time period.

DC, decompensated cirrhosis; HCC, hepatocellular carcinoma; HCV, Hepatitis C virus; AUD, Alcohol use disorder.

a Data from people in New South Wales, 1995–2022 (n = 64,865).

b Cumulative time contributed by individuals with HBV notifications until death or diagnosis of DC/HCC.

c Interquartile range.

d Among people with available information.

e Diagnosis of decompensated cirrhosis (DC) or hepatocellular carcinoma (HCC).

f Charlson comorbidity index score is an indicator of health; higher scores indicate worse health condition.

**Table 2:** Demographic characteristics of people with an HCV notification, by type of liver disease and time period.

	all HCV^a^	Period of DC and HCC diagnosis								
		2002–2007			2008–2014			2015–2022		
		all HCV	DC	HCC	all HCV	DC	HCC	all HCV	DC	HCC
Characteristics, n (%)	n = 112,277	n = 24,277	n = 1131	n = 288	n = 21,112	n = 2047	n = 888	n = 21,784	n = 2341	n = 1448
Person-years at risk^b^	1,748,365	341,639			859,731			1,549,430		
Year of birth, median (IQR)^c^^,^^d^	1967 (1959–1977)	1967 (1959–1976)	1956 (1949–1960)	1949 (1936–1956)	1971 (1961–1981)	1958 (1953–1963)	1955 (1948–1958)	1977 (1965–1988)	1961 (1956–1967)	1958 (1953–1962)
Age at diagnosis, median (IQR)^c^^,^^d^^,^^e^	55 (49–62)	55 (48–62)	49 (44–56)	55 (49–69)	56 (50–61)	53 (48–58)	57 (53–64)	58 (52–63)	57 (51–62)	60 (56–65)
Male sex^d^	72,047 (64)	14,983 (62)	824 (73)	228 (79)	13,557 (64)	1501 (73)	700 (79)	15,139 (69)	1708 (73)	1187 (82)
Female sex^d^	39,735 (35)	9218 (38)	305 (27)	60 (20)	7479 (35)	539 (26)	184 (21)	6570 (30)	625 (27)	258 (17)
HBV coinfection	4346 (4)	939 (4)	74 (7)	21 (7)	753 (4)	105 (5)	52 (6)	502 (2)	152 (7)	81 (6)
AUD	24,100 (22)	5547 (23)	646 (57)	66 (23)	5265 (25)	1264 (62)	375 (42)	4712 (22)	1497 (64)	716 (49)
Death	16,714 (14)	659 (3)	578 (51)	188 (66)	886 (4)	1193 (58)	577 (65)	1251 (6)	1502 (64)	926 (64)
Age at death, median (IQR)^c^^,^^d^	55 (46–64)	54 (45–63)	53 (47–61)	58 (51–73)	55 (45–63)	56 (51–61)	59 (54–66)	56 (44–64)	59 (53–64)	61 (57–66)
Charlson comorbidity index 3+^f^	10,013 (9)	2408 (10)	331 (29)	94 (33)	1805 (9)	626 (31)	318 (36)	1176	787 (34)	529 (37)

Demographic characteristics of people with an HCV notification, by type of liver disease and time period.

DC, decompensated cirrhosis; HCC, hepatocellular carcinoma; HBV, Hepatitis B virus, AUD, Alcohol use disorder.

a Data from people in New South Wales, 1995–2022 (n = 112,277).

b Cumulative time contributed by individuals with HCV notifications until death or diagnosis of DC/HCC.

c Interquartile range.

d Among people with available information.

e Diagnosis of decompensated cirrhosis (DC) or hepatocellular carcinoma (HCC).

f Charlson comorbidity index score is an indicator of health; higher scores indicate worse health condition.

During 2002–2022, some demographic and risk characteristics among the HBV and HCV population with DC and HCC shifted (Table 1, Table 2). For HBV, median ages at HBV-DC and HBV-HCC diagnosis increased from 56 to 58 years in 2002–2007 to 63 and 64 years in 2015–2022. The percentage of HBV-DC cases with a CCI score of 3 or more increased from 42% in 2002–2007 to 52% in 2015–2022. History of AUD among HBV-DC (17–19%) and HBV-HCC (7–8%) cases were relatively stable. For HCV, median ages at HCV-DC and HCV-HCC diagnosis increased from 49 and 55 years in 2002–2007 to 57 and 60 years in 2015–2022. History of AUD increased among HCV-DC (57–64%) and HCV-HCC (23–49%) cases. The percentage of HCV-DC cases with a CCI score of 3 or more increased from 29% in 2002–2007 to 34% in 2015–2022.

### Decompensated cirrhosis diagnoses and incidence

**Fig. 1:**
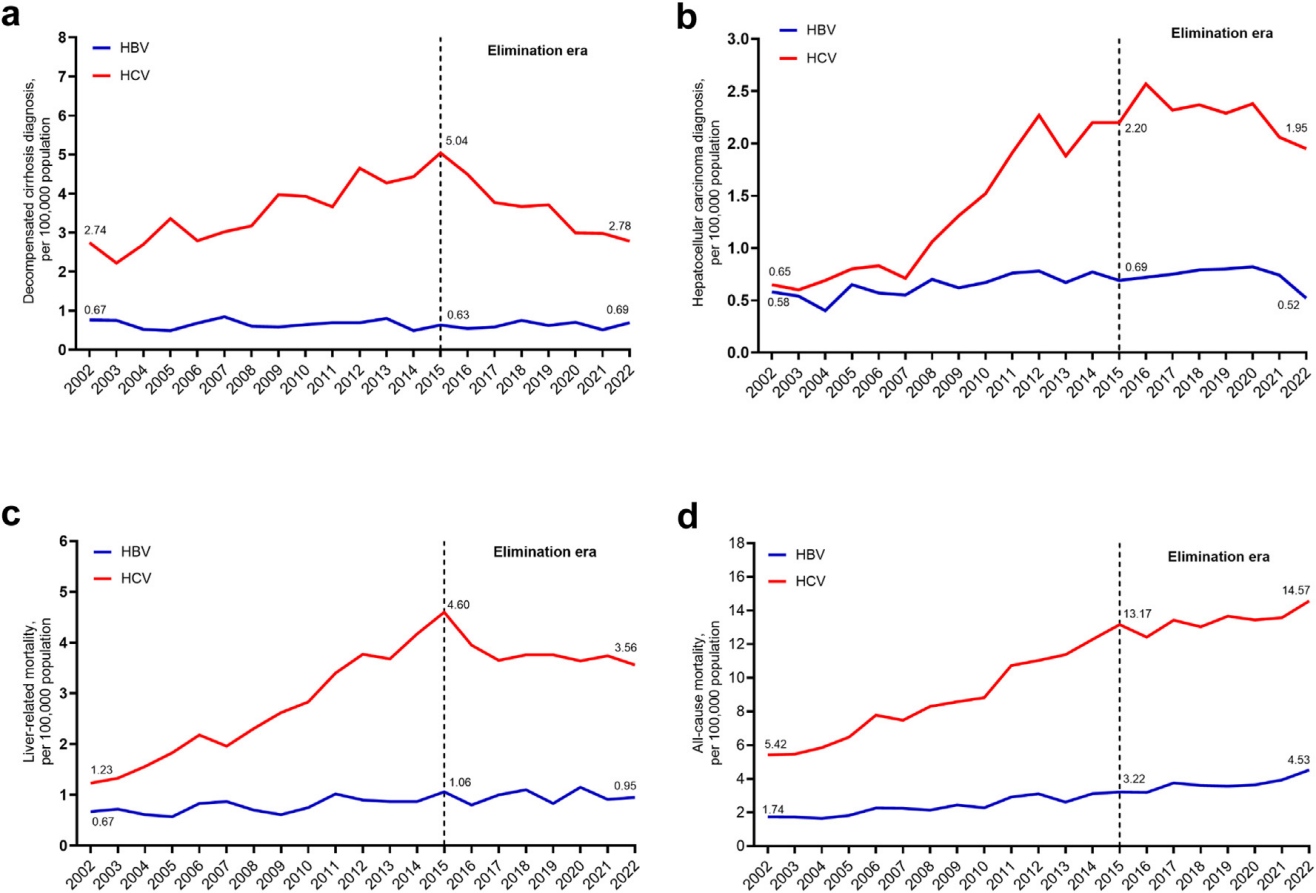
**Temporal trends in the numbers of (a) decompensated cirrhosis and (b) hepatocellular carcinoma diagnoses, (c) liver-related mortality, and (d) all-cause mortality, among people with an HBV and HCV notification**. Temporal trends in the numbers of (a) decompensated cirrhosis and (b) hepatocellular carcinoma diagnoses, (c) liver-related mortality, and (d) all-cause mortality, among people with an HBV and HCV notification.Data from individuals with an HBV notification (n = 64,865) and HCV notification (n = 112,277) in New South Wales, 1995–2022. (a) Decompensated cirrhosis and (b) hepatocellular carcinoma diagnoses, (c) liver-related mortality and (d) all-cause mortality. HBV, hepatitis B virus; HCV, hepatitis C virus.

**Fig. 2:**
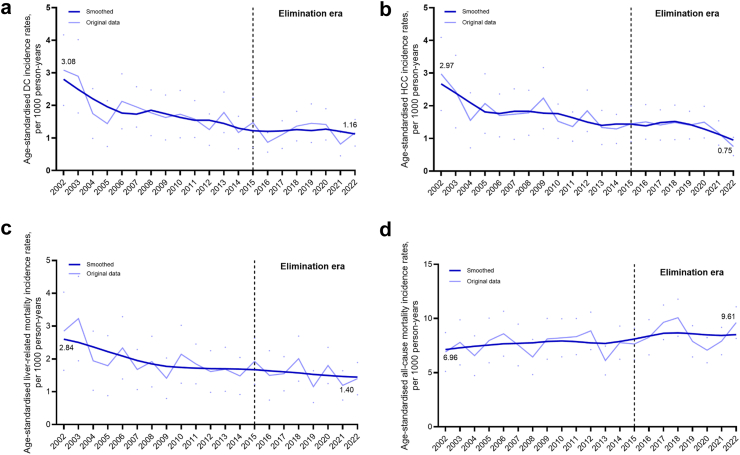
**Age-standardised (a) decompensated cirrhosis and (b) hepatocellular carcinoma diagnoses, (c) liver-related mortality, and (d) all-cause mortality incidence rates among people with an HBV notification**. Age-standardised (a) decompensated cirrhosis and (b) hepatocellular carcinoma diagnoses, (c) liver-related mortality, and (d) all-cause mortality incidence rates among people with an HBV notification. Data from people in New South Wales, 1995–2022 (n = 64,865). Age-standardised (a) decompensated cirrhosis and (b) hepatocellular carcinoma diagnoses, (c) liver-related mortality and (d) all-cause mortality incidence rates were calculated per 1000 person-years and corresponding 95% CIs (represented as dots in the panel figure) were calculated assuming a Poisson distribution. The Australian Standard Population 2013 was used for standardisation. DC, decompensated cirrhosis; HCC, hepatocellular carcinoma; HBV, hepatitis B virus.

**Fig. 3:**
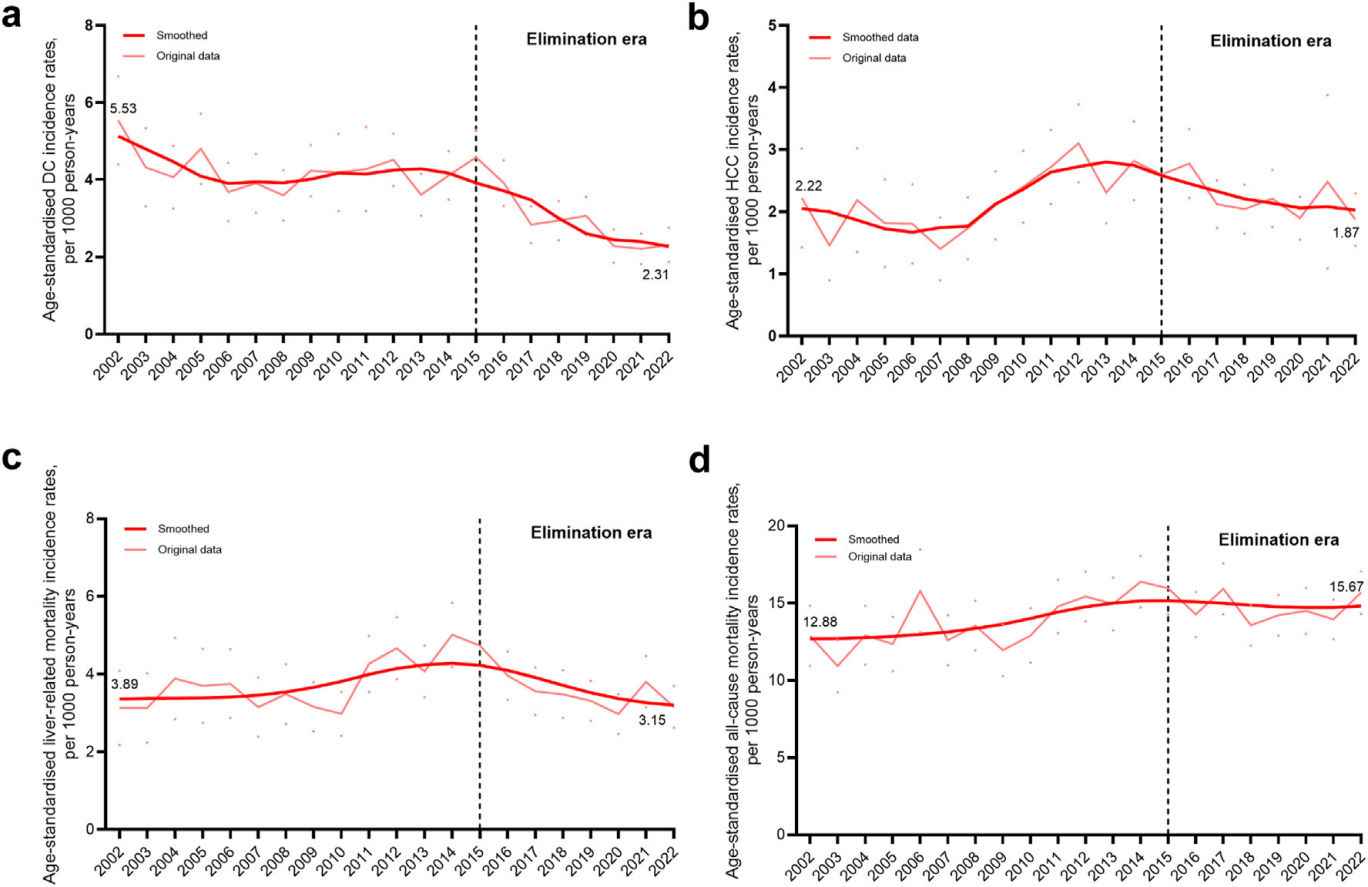
**Age-standardised (a) decompensated cirrhosis and (b) hepatocellular carcinoma diagnoses, (c) liver-related mortality, and (d) all-cause mortality incidence rates among people with HCV notification**. Age-standardised (a) decompensated cirrhosis and (b) hepatocellular carcinoma diagnoses, (c) liver-related mortality, and (d) all-cause mortality incidence rates among people with HCV notification. Data from people in New South Wales, 1995–2022 (n = 112,277). Age-standardised (a) decompensated cirrhosis and (b) hepatocellular carcinoma diagnoses, (c) liver-related mortality and (d) all-cause mortality incidence Rates were calculated per 1000 person-years and corresponding 95% CIs (represented as dots in the panel figure) were calculated assuming a Poisson distribution. The Australian Standard Population 2013 was used for standardisation. DC, decompensated cirrhosis; HCC, hepatocellular carcinoma; HCV, hepatitis C virus.

Annual HBV-DC diagnoses increased marginally from 44 (0.67 per 100,000 population) in 2002 to 57 (0.69 per 100,000) in 2022 (Fig. 1a, Table S4). Age-adjusted HBV-DC incidence declined progressively between 2002 and 2022 from 3.08 to 1.16 per 1000 PY (p < 0.001) (Fig. 2a). Annual HCV-DC diagnoses increased steadily from 181 (2.74 per 100,000) in 2002, peaking at 387 (5.04 per 100,000) in 2015 with a subsequent decline to 229 (2.78 per 100,000) in 2022 (Fig. 1a, Table S4). Age-adjusted HCV-DC incidence declined overall during the study period from 5.53 to 2.31 per 1000 PY, beginning with an initial decline from 2002 to 2006, followed by a period of relative stability from 2006 to 2015 (4.57 per 1000 PY in 2015), and then with a marked decline from 2015 to 2022 (2.31 per 1000 PY) (Fig. 3a).

Among people with HCV, those with a history of AUD had consistently higher annual HCV-DC diagnoses and age-adjusted incidence than those without AUD (Figure S1).

### Hepatocellular carcinoma diagnoses and incidence

Annual HBV-HCC diagnoses increased marginally from 38 (0.58 per 100,000 population) in 2002 to 53 (0.69 per 100,000) in 2015, then declined to 43 (0.52 per 100,000) in 2022 (p < 0.001) (Fig. 1b, Table S4). In contrast, age-adjusted HBV-HCC incidence declined progressively between 2002 and 2022 from 2.97 to 0.75 per 1000 PY (p < 0.001) (Fig. 2b). Annual HCV-HCC diagnoses increased markedly from 43 (0.65 per 100,000 population) in 2002 to 169 (2.20 per 100,000) in 2015, then declined to 161 (1.95 per 100,000) in 2022 (Fig. 1b, Table S4). Age-adjusted HCV-HCC incidence varied, with a stable rate 2002 to 2007, then increased 2008 to 2013, followed by steady decline during elimination era: 2.59 per 1000 PY in 2015 to 1.87 per 1000 PY in 2022 (Fig. 3b).

Among people with HCV, those with a history of AUD had consistently higher age-adjusted HCV-HCC incidence than those without AUD, although the incidence gap was narrower from 2015 (Figure S1).

### Mortality among people with HBV

Annual liver-related deaths among people with HBV increased from 44 (0.67 per 100,000 population) in 2002 to 81 (1.06 per 100,000) in 2015 then relatively stable, with 78 (0.95 per 100,000) in 2022 (p < 0.001) (Fig. 1c, Table S4). Age-adjusted incidence of liver-related mortality declined steadily between 2002 and 2022 from 2.84 to 1.40 per 1000 PY (p < 0.001) (Fig. 2c). Annual deaths due to any cause (all-cause mortality) among people with HBV increased from 115 (1.74 per 100,000 population) in 2002 to 374 (4.53 per 100,000) in 2022 (p < 0.001) (Fig. 1d, Table S4). Similarly, age-adjusted all-cause mortality increased between 2002 and 2022 from 6.91 to 9.62 per 1000 PY (p < 0.001) (Fig. 2d). Among people with HBV, liver-related and all-cause mortality for 2022 was 0.95 and 4.53 per 100,000 population, respectively (Fig. 2c and d).

### Mortality among people with HCV

Annual liver-related deaths among people with HCV increased from 81 (1.23 per 100,000 population) in 2002 to 353 (4.60 per 100,000) in 2015, then declined to 293 (3.56 per 100,000) in 2022 (Fig. 1c). Age-adjusted liver-related mortality increased from 3.89 per 1000 PY in 2002 to 4.73 per 1000 PY in 2015, then declined to 3.16 per 1000 PY in 2022 (Fig. 3c). Annual all-cause deaths among people with HCV increased markedly from 357 (5.42 per 100,000) in 2002 to 1200 (14.57 per 100,000) in 2022 (p < 0.001) (Fig. 1d). Age-adjusted all-cause mortality increased from 12.88 to 15.97 per 1000 PY in 2015, then was stable through 2022 (15.67 per 1000 PY) (Fig. 3d). Among people with HCV, liver-related and all-cause mortality in 2022 was 3.56 and 14.57 per 100,000 population, respectively (Fig. 2c and d).

### Impact of elimination era on DC and HCC, liver-related, and all-cause mortality, among people with HBV

In the period prior to the viral hepatitis elimination era (2002–2014), among people with HBV there was an increasing trend in diagnoses of DC, HCC, liver-related and all-cause mortality (count ratios [CR] for each 6-month interval: 1.00 [95% CI 1.00–1.01], 1.01 [95% CI 1.01–1.02], 1.01 [95% CI 1.00–1.02], and 1.03 [95% CI 1.02–1.03], respectively) (Table S5). The slope changes from the pre-elimination era to elimination era and within the elimination era demonstrate that these increases have been moderated, with less steep rises for DC, HCC, and liver-related mortality (Table S5). The exception is all-cause mortality which continues an upward trend over the elimination era (CR over each 6-month interval: 1.02 [95% CI 1.01–1.03]) (Table S4).

**Fig. 4:**
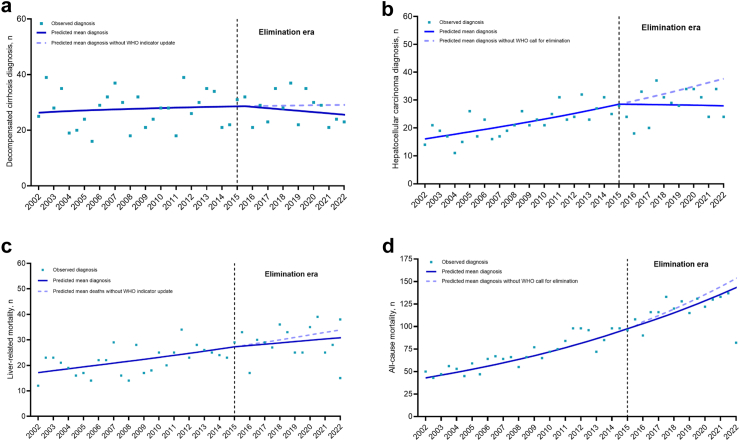
**Impact of the elimination era on numbers of (a) decompensated cirrhosis and (b) hepatocellular carcinomas diagnoses, (c) liver-related mortality, and (d) all-cause mortality, among people with an HBV notification**. Impact of the elimination era on numbers of (a) decompensated cirrhosis and (b) hepatocellular carcinomas diagnoses, (c) liver-related mortality, and (d) all-cause mortality, among people with an HCV notification. Data from individuals with an HBV notification in New South Wales, 1995–2022 (n = 64,865). (a) Decompensated cirrhosis and (b) hepatocellular carcinoma diagnoses, (c) liver-related mortality and (d) all-cause mortality. Segmented Poisson regression models, fitting a second time trend parameter using splines, were used to evaluate the effect of the elimination era on the numbers of decompensated cirrhosis and hepatocellular carcinoma diagnoses, liver-related mortality, and all-cause mortality. DC, decompensated cirrhosis; HBV, hepatitis B virus; HCC, hepatocellular carcinoma.

When the observed numbers were compared to those projected by the model, it was estimated that during the elimination era, 20 diagnoses of HBV-DC, 64 diagnoses of HBV-HCC, 44 liver-related deaths, and 122 all-cause deaths were prevented (Fig. 4a–d).

### Impact of elimination era on DC and HCC, liver-related, and all-cause mortality, among people with HCV

In the period prior to the viral hepatitis elimination era (2002–2014), among people with HCV there was a consistent increase in diagnoses of DC, HCC, and liver-related and all-cause mortality (CR for each 6-month interval: 1.03 [95% CI 1.02–1.04], 1.06 [95% CI 1.05–1.07], 1.05 [95% CI 1.04–1.06], and 1.05 [95% CI 1.04–1.06]), respectively (Table S5). Downward trends in liver-related morbidity and mortality were observed in the elimination era. This is reflected in the notable shifts observed in their respective trends, as indicated by a reduction in their slopes: 0.94 for DC, 0.93 for HCC, 0.94 for liver-related mortality, and 0.97 for all-cause mortality (Table S5). This translated to declining CRs for HCC-DC HCV-HCC liver-related mortality during the elimination era (CR for each 6-month interval: 0.96 [95% CI 0.95–0.97], 0.99 [95% CI 0.98–0.99], and 0.98 [95% CI 0.97–0.99]), respectively.

**Fig. 5:**
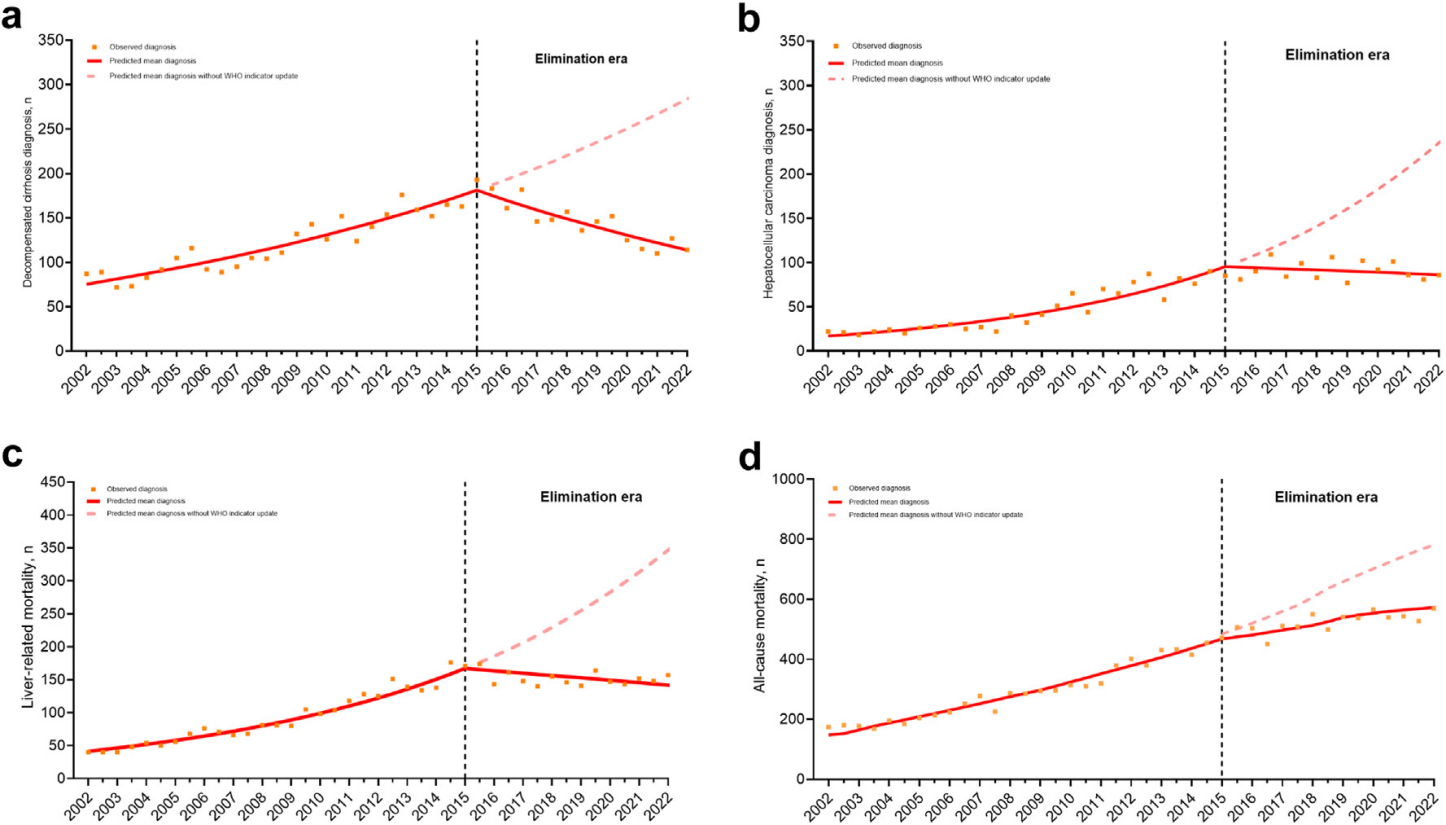
**Impact of the elimination era on numbers of (a) decompensated cirrhosis and (b) hepatocellular carcinomas diagnoses, (c) liver-related mortality, and (d) all-cause mortality, among people with an HCV notification**. Impact of the elimination era on numbers of (a) decompensated cirrhosis and (b) hepatocellular carcinomas diagnoses, (c) liver-related mortality, and (d) all-cause mortality, among people with an HCV notification. Data from individuals with an HCV notification in New South Wales, 1995–2022 (n = 112,277). (a) Decompensated cirrhosis diagnoses, (b) hepatocellular carcinoma diagnoses, (c) liver-related mortality and (d) all-cause mortality. Segmented Poisson regression models, fitting a second time trend parameter using splines, were used to evaluate the effect of the elimination era on the numbers of decompensated cirrhosis and hepatocellular carcinoma diagnoses, liver-related mortality, and all-cause mortality. DC, decompensated cirrhosis; HCC, hepatocellular carcinoma; HCV, hepatitis C virus.

When the observed numbers were compared to those projected by the model, it was estimated that in the elimination era, 1258 diagnoses of DC, 969 diagnoses of HCC, 1427 liver-related deaths, and 1648 all-cause deaths were prevented (Fig. 5a–d).

### Factors associated with liver-related mortality among people with HBV

The unadjusted and adjusted factors associated with liver-related mortality among people with HBV are presented in Table 3. In adjusted analyses, liver-related mortality was associated with older age (year of birth earlier than 1967) (adjusted incidence rate ratio (aIRR) 6.32; 95% CI 5.52–7.25), males (aIRR 2.28; 95% CI 2.04–2.57), CCI scores of 3 or greater (aIRR 1.62; 95% CI 1.39–1.89), and history of AUD (aIRR 4.59; 95% CI 4.00–5.25). Incidence of liver-related mortality declined in the elimination era (aIRR 0.68; 95% CI 0.59–0.80) (Table 3).

**Table 3:** Unadjusted and adjusted factors associated with liver-related mortality among people with an HBV notification.

Characteristics	Liver-related mortality n (%)	IRR	95% CI	p	aIRR	95% CI	p
Year of birth^a^							
≥1967	250 (<1)	1.00	–	–	1.00	–	–
<1967	1408 (5)	7.39	6.46–8.44	<0.001	6.32	5.52–7.25	<0.001
Sex							
Female	373 (1)	1.00	–	–	1.00	–	–
Male	1285 (4)	2.86	2.55–3.21	<0.001	2.28	2.04–2.57	<0.001
History of AUD							
No	1447 (2)	1.00	–	–	1.00	–	–
Yes	211 (14)	6.08	5.31–6.96	<0.001	4.59	4.00–5.25	<0.001
Charlson comorbidity index 3+^b^							
No	1487 (2)	1.00	–	–	1.00	–	–
Yes	171 (7)	2.86	2.45–3.33	<0.001	1.62	1.39–1.89	0.017
Elimination era^c^							
No	1481 (3)	1.00	–	–	1.00	–	–
Yes	177 (1)	0.46	0.39–0.54	<0.001	0.68	0.59–0.80	<0.001

Unadjusted and adjusted factors associated with liver-related mortality among people with an HBV notification. Data from people with an HBV notification (n = 64,865) in New South Wales, 1995–2022.

Factors associated with liver-related mortality were evaluated using unadjusted and adjusted Poisson regression analyses. The adjusted model is based on 64,555 individuals. aIRR, adjusted incidence rate ratio; HCV, hepatitis C virus; IRR, incidence rate ratio; AUD, Alcohol use disorder.

a Stratified around median.

b Charlson comorbidity index score is an indicator of health; higher scores indicate worse health condition.

c Elimination Era: Post 1 January 2015.

### Factors associated with liver-related mortality among people with HCV

The unadjusted and adjusted factors associated with liver-related mortality among those with HCV notification are presented in Table 4. In adjusted analyses, liver-related mortality was associated with older age (year of birth earlier than 1967– aIRR 7.14; 95% CI 6.56–7.77), males (aIRR 1.36; 95% CI 1.27–1.45), CCI scores of 3 or greater (aIRR 1.39; 95% CI 1.28–1.53), and history of AUD (aIRR 5.01; 95% CI 4.72–5.31). The incidence of liver-related mortality significantly declined in the elimination era (aIRR 0.38; 95% CI 0.21–0.27) (Table 4).

**Table 4:** Unadjusted and adjusted factors associated with liver-related mortality among people with an HCV notification.

Characteristics	Liver-related mortality n (%)	IRR	95% CI	p	aIRR	95% CI	p
Year of birth^a^							
≥1967	642 (1)	1.00	–	–	1.00	–	–
<1967	4167 (8)	7.24	6.66–7.86	<0.001	7.14	6.56–7.77	<0.001
Sex							
Female	1185 (3)	1.00	–	–	1.00	–	–
Male	3624 (5)	1.71	1.60–1.82	<0.001	1.36	1.27–1.45	<0.001
HBV co-infection							
No	4522 (4)	1.00	–	–	1.00	–	–
Yes	287 (7)	1.57	1.41–1.77	<0.001	1.40	1.24–1.58	<0.001
History of AUD							
No	2125 (2)	1.00	–	–	1.00	–	–
Yes	2684 (11)	4.62	4.37–4.88	<0.001	5.01	4.72–5.31	<0.001
Charlson comorbidity index 3+^b^							
No	4239 (4)	1.00	–	–	1.00	–	–
Yes	570 (9)	2.47	2.31–2.65	<0.001	1.39	1.28–1.53	<0.001
Elimination era^c^							
No	4549 (5)	1.00	–	–	1.00	–	–
Yes	260 (1)	0.24	0.21–0.27	<0.001	0.38	0.33–0.43	<0.001

Unadjusted and adjusted factors associated with liver-related mortality among people with an HCV notification. Data from people with an HCV notification (n = 112,277) in New South Wales, 1995–2022.

Factors associated with liver-related mortality were evaluated using unadjusted and adjusted Poisson regression analyses. The adjusted model is based on 111,782 individuals. aIRR, adjusted incidence rate ratio; HBV, hepatitis C virus; IRR, incidence rate ratio; AUD, Alcohol use disorder.

a Stratified around median.

b Charlson comorbidity index score is an indicator of health; higher scores indicate worse health condition.

c Elimination Era: Post 1 January 2015.

## Discussion

The response to HBV and HCV has been underpinned in recent years by a Global Health Sector Strategy to eliminate viral hepatitis as a public health threat by 2030 with associated WHO-established elimination targets.^2^ In this context, population-level evidence on HBV and HCV morbidity and mortality trends is critical. Our study, based on NSW linked administrative data, supports 2015 as a viral hepatitis inflection point in Australia: rising morbidity and mortality followed by turnaround in key outcome measures, particularly for HCV. The elimination era demonstrated reductions in HBV- and HCV-related advanced liver disease events (DC and HCC) and liver-related deaths comparing predicted (on basis of prior trends) to observed cases. The larger number of averted cases for HCV compared to HBV reflects both higher chronic infection prevalence,^27^^,^^28^ and more dramatic effect of rapid scale-up of DAA therapy. These results support the impact of a call for viral hepatitis elimination and are testament to Australia's public health approach to antiviral therapies for HBV and HCV, including a focus on equitable access.

Declining HBV-related morbidity and mortality in the elimination era is encouraging, given major advances in HBV therapy occurred in the mid-2000s (entecavir and tenofovir) contributing to improved clinical management^8^; but no new therapies since 2007. The continued declines in individual risk of DC, HCC, and liver-related death (as reflected by trends in age-adjusted incidence) could be explained through increasing HBV treatment coverage,^11^ enhanced HCC screening with earlier HCC diagnosis, and improved HCC management.^29^^,^^30^

In contrast, more dramatic declines in population burden and individual risk of HCV-related DC, HCC, and liver-related deaths from 2015 closely align with DAA introduction in Australia—first available to those with advanced liver disease through compassionate access mechanisms from late 2014, and then through government subsidy from March 2016.^22^ These results further highlight the role these therapies have played in reducing morbidity and mortality associated with HCV, concordant with results observed elsewhere,^31^, ^32^, ^33^ and relatively equitable access the Australian Government DAA program has enabled.

Australia has an ageing population of people with HBV and HCV, contributing to the pre-elimination era upward trajectory of HBV and HCV morbidity and mortality, and predictions of further increases.^34^, ^35^, ^36^ These predictions were due to increasing age-related risk of HBV- and HCV-related liver disease complications and also extra-hepatic complications that may be associated with mortality.^37^ Despite these predictions, we observed a significant turnaround corroborated by research from other settings.^32^^,^^38^, ^39^, ^40^ Notably, a study from Taiwan attributes a substantial decline of HCC mortality over four decades to population-wide intervention programs, including universal HBV vaccination and national viral hepatitis therapy.^41^ Furthermore, investigations in Scotland reported similar reductions in HCV- DC,^31^ liver-related and all-cause mortality,^33^ following the introduction of DAA.

The predicted HCV liver-related mortality, based on pre-2015 trends, with a more than three-fold increase in deaths in the period 2015–2022 reflects the ageing population and demonstrates the timeliness of DAA introduction. It also indicates why revised WHO mortality targets (from 65% reduction 2015–2030 to combined HBV and HCV mortality of ≤6/100,000/year)^3^ is more logical, particularly for settings where age distributions indicated increasing mortality for decades. Comparing predicted to observed liver-related deaths would have translated to a more than 65% reduction by 2022, but with 2015 set as baseline comparator the reduction is considerably more modest. Furthermore, prior Australian HCV modelling has demonstrated that despite impressive DAA uptake, a 65% mortality reduction was not achievable.^13^ Current combined liver-related mortality from HCV (3.56/10,000/year) and HBV (0.95/100,000) is already below the WHO elimination target. Despite this, Australia's 6th National Hepatitis C Strategy has established a 2030 target of ≤1/100,000/year,^42^ to highlight the need to continue the momentum.

Our findings also highlight the importance of addressing liver disease co-morbidities. The decline in age-standardised HCV-DC incidence among those with AUD, from 17.9/1000 py in 2015 to 6.5/1000 in 2022 was dramatic, potentially indicating a combined effect of HCV cure and reductions in alcohol use, highlighting the benefit of interventions to address AUD while concomitantly treating HCV.^43^^,^^44^ People with AUD represent a critical intersection where lifestyle factors and infectious diseases converge, escalating risk of severe liver complications.^44^ A recent Danish study found that patients with alcohol-related liver disease are at high risk of liver-related mortality in the first 5 years after diagnosis.^45^ Similarly, patients notified with HBV or HCV and having a CCI score of 3 or higher, who face a twofold increased relative risk of liver-related mortality, highlighting the impact of multiple comorbidities in these populations.^46^^,^^47^ Our study reiterates the necessity of strategies that not only cure HCV but also address lifestyle factors that may increase risk of morbidity and mortality.

While our overall study findings are encouraging and indicate progress towards elimination in NSW, modelling has suggested that Australia may not be on track to meet previous WHO elimination targets,^13^ nor all revised absolute targets by 2030.^14^ For HBV elimination in Australia, bridging the gap for people diagnosed with chronic HBV who are not engaged with care, reducing late HBV diagnoses, targeting at-risk populations (migrant, culturally and linguistically diverse, Aboriginal and Torres Strait Islander peoples) are identified as key areas of interest.^48^ For HCV, demonstrated strategies to enhance linkage to care will be crucial to achieving elimination, including integrated care, point-of-care testing, and expanding DAA prescribing to pharmacists.^49^ While Australia was early to expand DAA prescribing, prescriber restrictions have been recently identified as the most common DAA restriction globally, which inadvertently becomes a major barrier for at-risk populations who seek care.^50^ These are critical system-level changes essential to achieve viral hepatitis elimination.

There are several limitations to this study that should be acknowledged. Firstly, our study relies on laboratory-based HCV notifications in NSW following HCV antibody diagnosis. Consequently, a proportion (estimated at 25% for men; 34% for women) of these notifications could correspond to cases with undetectable HCV RNA at the time of diagnosis; an indication of potential spontaneous clearance.^51^ Secondly, the process for identifying liver-related mortality, based on hospitalisation data for DC or HCC, inherently excludes liver-related deaths that occur without hospital admission. While this method has its own set of limitations in accurately determining the cause of death and risk of overestimation, it has been validated in NSW for both HBV and HCV.^18^ Lastly, an assessment of the impact of the elimination era on cause-specific, non-liver related mortality was not conducted. Results from our study for all-cause HCV-related mortality suggest an additional non-hepatic benefit contributing toward reductions in all-cause mortality. As such, an examination of specific subpopulations and their cause-specific mortality at risk is warranted and will be the focus of our next study.

In conclusion, this study provides evidence of declining risk of HBV and HCV related morbidity and mortality, suggesting the impact of the call for viral hepatitis elimination and the therapeutic advancements and effective clinical management for both HBV and HCV. Further strategies to reduce the burden of viral hepatitis include enhanced screening and earlier diagnosis, improved treatment coverage, and addressing modifiable co-factors such as AUD.

## Contributors

ST, HV, SAP and GD contributed to study conception and design, data analysis and interpretation of the findings. ST and HV both verified the data and had access to raw data. Shane Tillakeratne drafted the original manuscript under the supervision of HV, SAP and GD. ST had final responsibility for the decision to submit for publication. All authors contributed towards writing of and approving the final manuscript.

## Data sharing statement

This publication involved information collected by population-based health administration registries. Data used for this research cannot be deposited on servers other than those approved by Ethics Committees. This publication has used highly sensitive health information through linkage of several administrative datasets. De-identified linked information has been provided to the research team under strict privacy regulations. Except in the form of conclusions drawn from the data, researchers do not have permission to disclose any data to any person other than those authorised for the research project.

## Declaration of interests

GD reports research support from Gilead and Abbvie. HV has received honoraria from Gilead Sciences. JGe received consulting fees from NovoNordisk, participated on a Data Safety Monitoring Board for AbbVie, Gilead Sciences, BMS, Pharmaxis, Novartis, Cincera, Pfizer, Roche, NovoNordisk, Eisai and Bayer. JGr has received research grants from AbbVie, Biolytical, Cepheid, Gilead and Hologic, and has received honoraria from AbbVie, Abbott, Cepheid, Gilead and Roche outside the submitted work. GM reports grants from ViiV and Janssen, received honororia from ViiV and Gilead and participated on a Data Safety Monitoring Board for ViiV. All remaining authors have no potential conflicts to declare. Disclaimer: All inferences, opinions, and conclusions drawn in this publication are those of the author(s), and do not necessarily reflect the opinions or policies of the Australian Government Department of Health.
