# Supplementary material for: Trends in viral hepatitis liver-related morbidity and mortality in New South Wales, Australia
Source: Lancet Reg Health West Pac. 2024 Aug 31;51:101185. doi: 10.1016/j.lanwpc.2024.101185 (PMC11402402; doi:10.1016/j.lanwpc.2024.101185)
Supplement: Figure S1 [file mmc1.docx]

**Supplementary Figure 1. Temporal trends in (a) decompensated cirrhosis and (b) age-standardised DC incidence rates, (c) hepatocellular carcinoma and (d) age-standardised HCC incidence rates among people with an HCV notification, by alcohol-use disorder.**


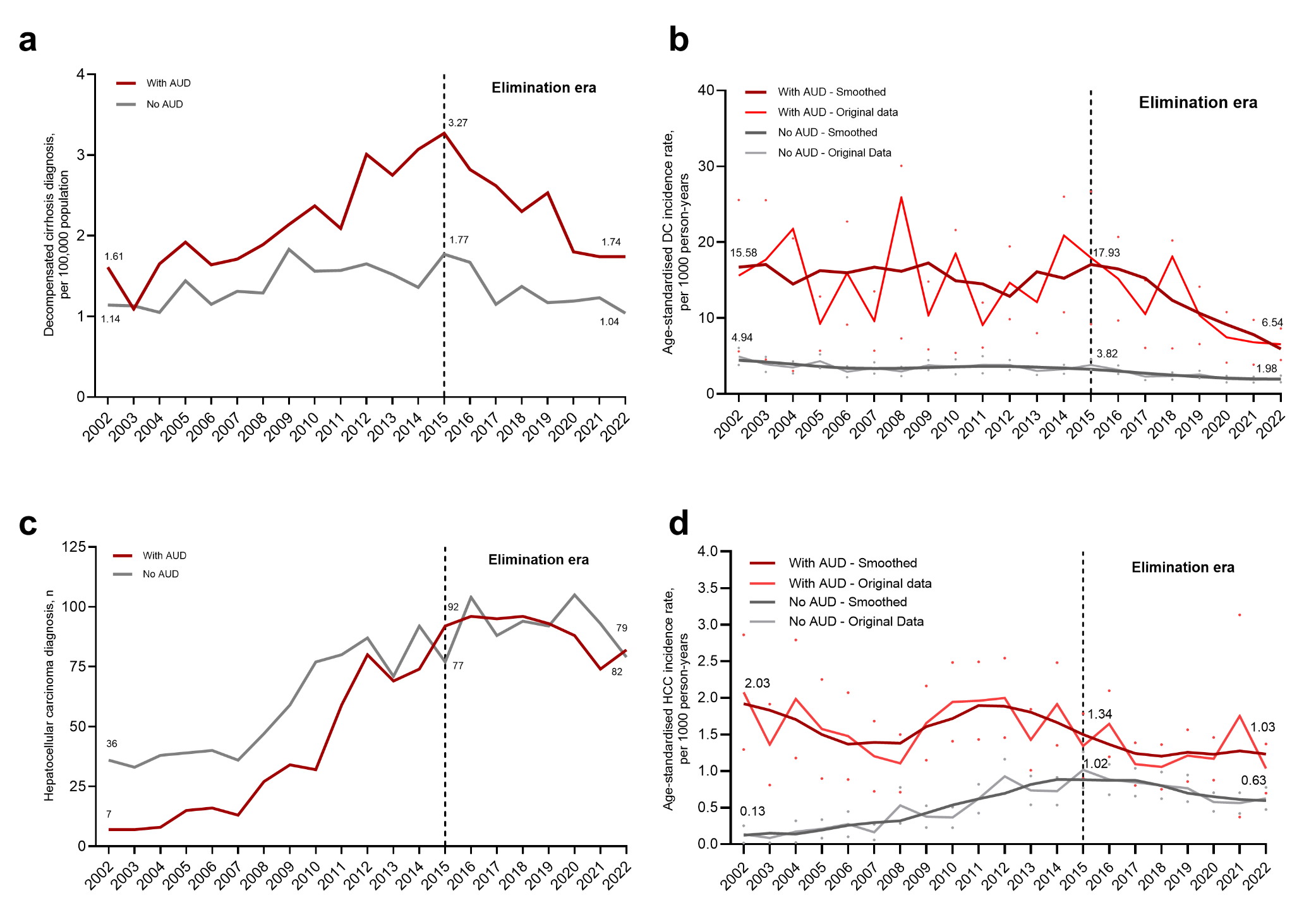


Temporal trends in (a) decompensated cirrhosis and (b) age-standardised DC incidence rates, (c) hepatocellular carcinoma and (d) age-standardised HCC incidence rates among people with an HCV notification, by alcohol-use disorder. Data from people in New South Wales, by alcohol-use disorder, 1995–2022 (n = 112,277). Age-standardised decompensated cirrhosis and hepatocellular carcinoma incidence rates were calculated per 1000 person-years and corresponding 95% CIs were calculated assuming a Poisson distribution. The Australian Standard Population 2013 was used for standardisation. DC, decompensated cirrhosis; HCC, hepatocellular carcinoma; HCV, hepatitis C virus.
