# Supplementary material for: Trends in viral hepatitis liver-related morbidity and mortality in New South Wales, Australia
Source: Lancet Reg Health West Pac. 2024 Aug 31;51:101185. doi: 10.1016/j.lanwpc.2024.101185 (PMC11402402; doi:10.1016/j.lanwpc.2024.101185)
Supplement: Table S1 [file mmc2.docx]

**Supplementary Table 1. Set of relevant ICD-10 codes for decompensated cirrhosis (DC)**

| **Inferred Diagnosis** | **ICD-10 Code** | **DC-related hospital admission** |
| --- | --- | --- |
| **DC** | R18 | Ascites |
|  | I85.0 | Oesophageal varices |
|  | I98.3 | Oesophageal varices with bleeding in diseases classified elsewhere |
|  | K72.1 | Chronic hepatic failure |
|  | K72.9 | Hepatic failure, unspecified |
|  | K70.4 | Alcoholic hepatic failure. |
|  | K76.7 | Hepatorenal syndrome |

Set of relevant ICD-10 codes for decompensated cirrhosis (DC).
