# Supplementary material for: Trends in viral hepatitis liver-related morbidity and mortality in New South Wales, Australia
Source: Lancet Reg Health West Pac. 2024 Aug 31;51:101185. doi: 10.1016/j.lanwpc.2024.101185 (PMC11402402; doi:10.1016/j.lanwpc.2024.101185)
Supplement: Table S2 [file mmc3.docx]

**Supplementary Table 2. Set of relevant ICD-10 codes for alcohol-use disorder (AUD)**

| **Inferred Diagnosis** | **ICD-10 Code** | **AUD-related hospital admission** |
| --- | --- | --- |
| **AUD** | E24.4 | Alcohol induced Pseudo-Cushing’s syndrome |
|  | F10 | Mental and behavioural disorders due to use of alcohol |
|  | G31.2 | Degeneration of nervous system due to alcohol |
|  | G62.1 | Alcoholic polyneuropathy |
|  | I42.6 | Alcoholic cardiomyopathy |
|  | G72.1 | Alcoholic myopathy |
|  | Z50.2 | Alcohol rehabilitation |
|  | Z71.4 | Alcohol abuse counselling and surveillance |

Set of relevant ICD-10 codes for alcohol-use disorder (AUD).
