# Supplementary material for: Trends in viral hepatitis liver-related morbidity and mortality in New South Wales, Australia
Source: Lancet Reg Health West Pac. 2024 Aug 31;51:101185. doi: 10.1016/j.lanwpc.2024.101185 (PMC11402402; doi:10.1016/j.lanwpc.2024.101185)
Supplement: Table S3 [file mmc4.docx]

**Supplementary Table 3.** **Demographic characteristics of people with an HBV/HCV notification by sex, (2002-2022)**

|  | **HBV** | | **HCV** | |
| --- | --- | --- | --- | --- |
|  | **Male** | **Female** | **Male** | **Female** |
| Characteristics, n (%) | n=35,262 | n=29,290 | n=72,047 | n=39,735 |
| Person-years at risk | 539,668 | 456,907 | 1,090,275 | 649,082 |
| Year of birth, median (IQR)^ab^ | 1968 (1957-1979) | 1971 (1960-1981) | 1967 (1959-1977) | 1968 (1959-1978) |
| Age of diagnosis, median (IQR)^abc^ | 59 (51-68) | 60 (51-69) | 55 (49-61) | 55 (48-65) |
| AUD | 1,177 (3) | 335 (1) | 17,454 (24) | 6,597 (17) |
| Death | 2,587 (7) | 1,149 (4) | 11,926 (17) | 4,752 (12) |
| Age at death, median (IQR)^ab^ | 66 (55-77) | 68 (56-79) | 55 (46-62) | 56 (46-71) |
| Charlson comorbidity index 3+^e^ | 2,368 (7) | 1,400 (5) | 6,628 (9) | 3,360 (9) |

Demographic characteristics of people with an HBV/HCV notification by sex, (2002-2022). Data from people in New South Wales, 1995–2022 (HBV: n=64,865; HCV: n=112,277)

^a^ Interquartile range.

^b^ Among people with available information.

^c^ Diagnosis of decompensated cirrhosis or hepatocellular carcinoma (HCC)

^d^ Charlson comorbidity index score is an indicator of health; higher scores indicate worse health condition.

DC, decompensated cirrhosis; HCC, hepatocellular carcinoma; HBV, Hepatitis B virus, AUD, Alcohol use disorder.
