# Supplementary material for: Trends in viral hepatitis liver-related morbidity and mortality in New South Wales, Australia
Source: Lancet Reg Health West Pac. 2024 Aug 31;51:101185. doi: 10.1016/j.lanwpc.2024.101185 (PMC11402402; doi:10.1016/j.lanwpc.2024.101185)
Supplement: Table S4 [file mmc5.docx]

**Supplementary Table 4. Numbers of decompensated cirrhosis and hepatocellular carninoma diagnoses, all-cause and liver related mortality among people with an HBV/HCV notification, (2002-2022).**

| **Year** | **HBV** | | | | **HCV** | | | |
| --- | --- | --- | --- | --- | --- | --- | --- | --- |
|  | **Decompensated cirrhosis** | **Hepatocellular carcinoma** | **Liver-related mortality** | **All-cause mortality** | **Decompensated cirrhosis** | **Hepatocellular carcinoma** | **Liver-related mortality** | **All-cause mortality** |
| 2002 | 44 | 38 | 44 | 115 | 181 | 43 | 81 | 357 |
| 2003 | 50 | 36 | 48 | 115 | 147 | 40 | 88 | 363 |
| 2004 | 35 | 27 | 41 | 110 | 180 | 46 | 104 | 391 |
| 2005 | 33 | 44 | 38 | 122 | 226 | 54 | 123 | 435 |
| 2006 | 46 | 39 | 56 | 154 | 189 | 56 | 148 | 528 |
| 2007 | 58 | 38 | 60 | 155 | 208 | 49 | 135 | 515 |
| 2008 | 42 | 49 | 49 | 150 | 222 | 74 | 162 | 581 |
| 2009 | 41 | 44 | 43 | 174 | 282 | 93 | 186 | 609 |
| 2010 | 46 | 48 | 54 | 164 | 282 | 109 | 203 | 633 |
| 2011 | 50 | 55 | 74 | 212 | 266 | 139 | 247 | 779 |
| 2012 | 51 | 57 | 66 | 229 | 342 | 167 | 277 | 812 |
| 2013 | 60 | 50 | 65 | 195 | 318 | 140 | 274 | 848 |
| 2014 | 37 | 58 | 66 | 236 | 335 | 166 | 315 | 929 |
| 2015 | 48 | 53 | 81 | 247 | 387 | 169 | 353 | 1010 |
| 2016 | 42 | 56 | 62 | 249 | 350 | 200 | 308 | 968 |
| 2017 | 46 | 59 | 79 | 297 | 298 | 183 | 288 | 1061 |
| 2018 | 60 | 63 | 88 | 289 | 294 | 190 | 301 | 1044 |
| 2019 | 50 | 65 | 67 | 288 | 300 | 185 | 304 | 1106 |
| 2020 | 57 | 66 | 93 | 295 | 242 | 193 | 295 | 1089 |
| 2021 | 41 | 60 | 74 | 319 | 241 | 167 | 303 | 1100 |
| 2022 | 57 | 43 | 78 | 374 | 229 | 161 | 293 | 1200 |

Numbers of decompensated cirrhosis and hepatocellular carninoma diagnoses, all-cause and liver related mortality among people with an HBV/HCV notification, (2002-2022).
