# Supplementary material for: Trends in viral hepatitis liver-related morbidity and mortality in New South Wales, Australia
Source: Lancet Reg Health West Pac. 2024 Aug 31;51:101185. doi: 10.1016/j.lanwpc.2024.101185 (PMC11402402; doi:10.1016/j.lanwpc.2024.101185)
Supplement: Table S5 [file mmc6.docx]

**Supplementary Table 5. Impact of the elimination era on numbers of decompensated cirrhosis and hepatocellular carcinoma diagnoses, liver-related and all-cause mortality.**

|  | **Slope pre-call for elimination^a^ CR (95% CI)** | ***p*** | **Slope change CR (95%CI)** | ***p*** | **Slope post- call for elimination^b^ CR (95% CI)** | ***p*** |
| --- | --- | --- | --- | --- | --- | --- |
| **HBV** | | | | | | |
| Decompensated cirrhosis | 1·00 (1·00-1·01) | 0·854 | 0·99 (0·99-1·00) | 0·476 | 1·01 (0·99-1·03) | 0·295 |
| Hepatocellular carcinoma | 1·01 (1·01-1·03) | <0·001 | 0·97 (0·95-0·99) | 0·051 | 0·99 (0·98-1·01) | 0·629 |
| Liver-related mortality | 1·01 (1·00-1·02) | 0·005 | 0·99 (0·97-1·02) | 0·540 | 1·00 (0·99-1·02) | 0·423 |
| All-cause mortality | 1·03 (1·02-1·03) | <0·001 | 0·99 (0·98-1·00) | 0·718 | 1·02 (1·01- 1·03) | <0·001 |
| **HCV** | | | | | | |
| Decompensated cirrhosis | 1·03 (1·02-1·04) | <0·001 | 0·94 (0·92- 0·94) | <0·001 | 0·96 (0·95-0·97) | <0·001 |
| Hepatocellular carcinoma | 1·06 (1·05-1·07) | <0·001 | 0·93 (0·91-0·94) | <0·001 | 0·99 (0·98-0·99) | <0·041 |
| Liver-related mortality | 1·05 (1·04-1·06) | <0·001 | 0·94 (0·93-0·95) | <0·001 | 0·98(0·97-0·99) | <0·001 |
| All-cause mortality | 1·04 (1·03-1·04) | <0·001 | 0·97 (0·96-0·98) | <0·001 | 1·01(1·00-1·01) | <0·001 |

Impact of the elimination era on numbers of decompensated cirrhosis and hepatocellular carcinoma diagnoses, liver-related and all-cause mortality. Data from people with an HBV notification (n = 64,865) and HCV notification (n = 112,277) in New South Wales, 1995–2022.

^a^ Pre elimination era: 2002–2014.

^b^ Elimination era: 2015-2022.

Segmented Poisson regression models, fitting a second time trend parameter using splines, were used to evaluate the effect of the elimination era on the numbers of decompensated cirrhosis and hepatocellular carcinoma diagnoses, liver-related mortality, and all-cause mortality among people with an HBV and HCV notification. HCV, hepatitis C virus; CR, Count ratio.
