# Supplementary material for: Trends in viral hepatitis liver-related morbidity and mortality in New South Wales, Australia
Source: Lancet Reg Health West Pac. 2024 Aug 31;51:101185. doi: 10.1016/j.lanwpc.2024.101185 (PMC11402402; doi:10.1016/j.lanwpc.2024.101185)
Supplement: Supplementary material [file mmc7.docx]

Supplementary tables and figures

- Page 2 - Supplementary Table 1. Set of relevant ICD-10 codes for decompensated cirrhosis (DC)
- Page 3 - Supplementary Table 2. Set of relevant ICD-10 codes for alcohol-use disorder (AUD)
- Page 4 - Supplementary Table 3. Demographic characteristics of people with an HBV/HCV notification by sex, (2002-2022)
- Page 5 - Supplementary Table 4. Numbers of decompensated cirrhosis and hepatocellular carcinoma diagnoses, liver-related and all-cause mortality among people with an HBV/HCV notification, (2002-2022).
- Page 6 - Supplementary Table 5. Impact of the elimination era on numbers of decompensated cirrhosis and hepatocellular carcinoma diagnoses, liver-related and all-cause mortality.
- Page 7 - Supplementary Figure 1. Temporal trends in (A) decompensated cirrhosis and (B) age-standardised DC incidence rates, (C) hepatocellular carcinoma and (D) age-standardised HCC incidence rates among people with an HCV notification, by alcohol-use disorder.

**Supplementary Table 1. Set of relevant ICD-10 codes for decompensated cirrhosis (DC)**

| **Inferred Diagnosis** | **ICD-10 Code** | **DC-related hospital admission** |
| --- | --- | --- |
| **DC** | R18 | Ascites |
|  | I85.0 | Oesophageal varices |
|  | I98.3 | Oesophageal varices with bleeding in diseases classified elsewhere |
|  | K72.1 | Chronic hepatic failure |
|  | K72.9 | Hepatic failure, unspecified |
|  | K70.4 | Alcoholic hepatic failure. |
|  | K76.7 | Hepatorenal syndrome |

Set of relevant ICD-10 codes for decompensated cirrhosis (DC).

**Supplementary Table 2. Set of relevant ICD-10 codes for alcohol-use disorder (AUD)**

| **Inferred Diagnosis** | **ICD-10 Code** | **AUD-related hospital admission** |
| --- | --- | --- |
| **AUD** | E24.4 | Alcohol induced Pseudo-Cushing’s syndrome |
|  | F10 | Mental and behavioural disorders due to use of alcohol |
|  | G31.2 | Degeneration of nervous system due to alcohol |
|  | G62.1 | Alcoholic polyneuropathy |
|  | I42.6 | Alcoholic cardiomyopathy |
|  | G72.1 | Alcoholic myopathy |
|  | Z50.2 | Alcohol rehabilitation |
|  | Z71.4 | Alcohol abuse counselling and surveillance |

Set of relevant ICD-10 codes for alcohol-use disorder (AUD).

**Supplementary Table 3.** **Demographic characteristics of people with an HBV/HCV notification by sex, (2002-2022)**

|  | **HBV** | | **HCV** | |
| --- | --- | --- | --- | --- |
|  | **Male** | **Female** | **Male** | **Female** |
| Characteristics, n (%) | n=35,262 | n=29,290 | n=72,047 | n=39,735 |
| Person-years at risk | 539,668 | 456,907 | 1,090,275 | 649,082 |
| Year of birth, median (IQR)^ab^ | 1968 (1957-1979) | 1971 (1960-1981) | 1967 (1959-1977) | 1968 (1959-1978) |
| Age of diagnosis, median (IQR)^abc^ | 59 (51-68) | 60 (51-69) | 55 (49-61) | 55 (48-65) |
| AUD | 1,177 (3) | 335 (1) | 17,454 (24) | 6,597 (17) |
| Death | 2,587 (7) | 1,149 (4) | 11,926 (17) | 4,752 (12) |
| Age at death, median (IQR)^ab^ | 66 (55-77) | 68 (56-79) | 55 (46-62) | 56 (46-71) |
| Charlson comorbidity index 3+^e^ | 2,368 (7) | 1,400 (5) | 6,628 (9) | 3,360 (9) |

Demographic characteristics of people with an HBV/HCV notification by sex, (2002-2022). Data from people in New South Wales, 1995–2022 (HBV: n=64,865; HCV: n=112,277)

^a^ Interquartile range.

^b^ Among people with available information.

^c^ Diagnosis of decompensated cirrhosis or hepatocellular carcinoma (HCC)

^d^ Charlson comorbidity index score is an indicator of health; higher scores indicate worse health condition.

DC, decompensated cirrhosis; HCC, hepatocellular carcinoma; HBV, Hepatitis B virus, AUD, Alcohol use disorder.

**Supplementary Table 4. Numbers of decompensated cirrhosis and hepatocellular carninoma diagnoses, all-cause and liver related mortality among people with an HBV/HCV notification, (2002-2022).**

| Year | HBV | | | | HCV | | | |
| --- | --- | --- | --- | --- | --- | --- | --- | --- |
|  | Decompensated cirrhosis | Hepatocellular carcinoma | Liver-related mortality | All-cause mortality | Decompensated cirrhosis | Hepatocellular carcinoma | Liver-related mortality | All-cause mortality |
| 2002 | 44 | 38 | 44 | 115 | 181 | 43 | 81 | 357 |
| 2003 | 50 | 36 | 48 | 115 | 147 | 40 | 88 | 363 |
| 2004 | 35 | 27 | 41 | 110 | 180 | 46 | 104 | 391 |
| 2005 | 33 | 44 | 38 | 122 | 226 | 54 | 123 | 435 |
| 2006 | 46 | 39 | 56 | 154 | 189 | 56 | 148 | 528 |
| 2007 | 58 | 38 | 60 | 155 | 208 | 49 | 135 | 515 |
| 2008 | 42 | 49 | 49 | 150 | 222 | 74 | 162 | 581 |
| 2009 | 41 | 44 | 43 | 174 | 282 | 93 | 186 | 609 |
| 2010 | 46 | 48 | 54 | 164 | 282 | 109 | 203 | 633 |
| 2011 | 50 | 55 | 74 | 212 | 266 | 139 | 247 | 779 |
| 2012 | 51 | 57 | 66 | 229 | 342 | 167 | 277 | 812 |
| 2013 | 60 | 50 | 65 | 195 | 318 | 140 | 274 | 848 |
| 2014 | 37 | 58 | 66 | 236 | 335 | 166 | 315 | 929 |
| 2015 | 48 | 53 | 81 | 247 | 387 | 169 | 353 | 1010 |
| 2016 | 42 | 56 | 62 | 249 | 350 | 200 | 308 | 968 |
| 2017 | 46 | 59 | 79 | 297 | 298 | 183 | 288 | 1061 |
| 2018 | 60 | 63 | 88 | 289 | 294 | 190 | 301 | 1044 |
| 2019 | 50 | 65 | 67 | 288 | 300 | 185 | 304 | 1106 |
| 2020 | 57 | 66 | 93 | 295 | 242 | 193 | 295 | 1089 |
| 2021 | 41 | 60 | 74 | 319 | 241 | 167 | 303 | 1100 |
| 2022 | 57 | 43 | 78 | 374 | 229 | 161 | 293 | 1200 |

Numbers of decompensated cirrhosis and hepatocellular carninoma diagnoses, all-cause and liver related mortality among people with an HBV/HCV notification, (2002-2022).

**Supplementary Table 5. Impact of the elimination era on numbers of decompensated cirrhosis and hepatocellular carcinoma diagnoses, liver-related and all-cause mortality.**

|  | **Slope pre-call for elimination^a^ CR (95% CI)** | ***p*** | **Slope change CR (95%CI)** | ***p*** | **Slope post- call for elimination^b^ CR (95% CI)** | ***p*** |
| --- | --- | --- | --- | --- | --- | --- |
| **HBV** | | | | | | |
| Decompensated cirrhosis | 1·00 (1·00-1·01) | 0·854 | 0·99 (0·99-1·00) | 0·476 | 1·01 (0·99-1·03) | 0·295 |
| Hepatocellular carcinoma | 1·01 (1·01-1·03) | <0·001 | 0·97 (0·95-0·99) | 0·051 | 0·99 (0·98-1·01) | 0·629 |
| Liver-related mortality | 1·01 (1·00-1·02) | 0·005 | 0·99 (0·97-1·02) | 0·540 | 1·00 (0·99-1·02) | 0·423 |
| All-cause mortality | 1·03 (1·02-1·03) | <0·001 | 0·99 (0·98-1·00) | 0·718 | 1·02 (1·01- 1·03) | <0·001 |
| **HCV** | | | | | | |
| Decompensated cirrhosis | 1·03 (1·02-1·04) | <0·001 | 0·94 (0·92- 0·94) | <0·001 | 0·96 (0·95-0·97) | <0·001 |
| Hepatocellular carcinoma | 1·06 (1·05-1·07) | <0·001 | 0·93 (0·91-0·94) | <0·001 | 0·99 (0·98-0·99) | <0·041 |
| Liver-related mortality | 1·05 (1·04-1·06) | <0·001 | 0·94 (0·93-0·95) | <0·001 | 0·98(0·97-0·99) | <0·001 |
| All-cause mortality | 1·04 (1·03-1·04) | <0·001 | 0·97 (0·96-0·98) | <0·001 | 1·01(1·00-1·01) | <0·001 |

Impact of the elimination era on numbers of decompensated cirrhosis and hepatocellular carcinoma diagnoses, liver-related and all-cause mortality. Data from people with an HBV notification (n = 64,865) and HCV notification (n = 112,277) in New South Wales, 1995–2022.

^a^ Pre elimination era: 2002–2014.

^b^ Elimination era: 2015-2022.

Segmented Poisson regression models, fitting a second time trend parameter using splines, were used to evaluate the effect of the elimination era on the numbers of decompensated cirrhosis and hepatocellular carcinoma diagnoses, liver-related mortality, and all-cause mortality among people with an HBV and HCV notification. HCV, hepatitis C virus; CR, Count ratio.

**Supplementary Figure 1. Temporal trends in (a) decompensated cirrhosis and (b) age-standardised DC incidence rates, (c) hepatocellular carcinoma and (d) age-standardised HCC incidence rates among people with an HCV notification, by alcohol-use disorder.**


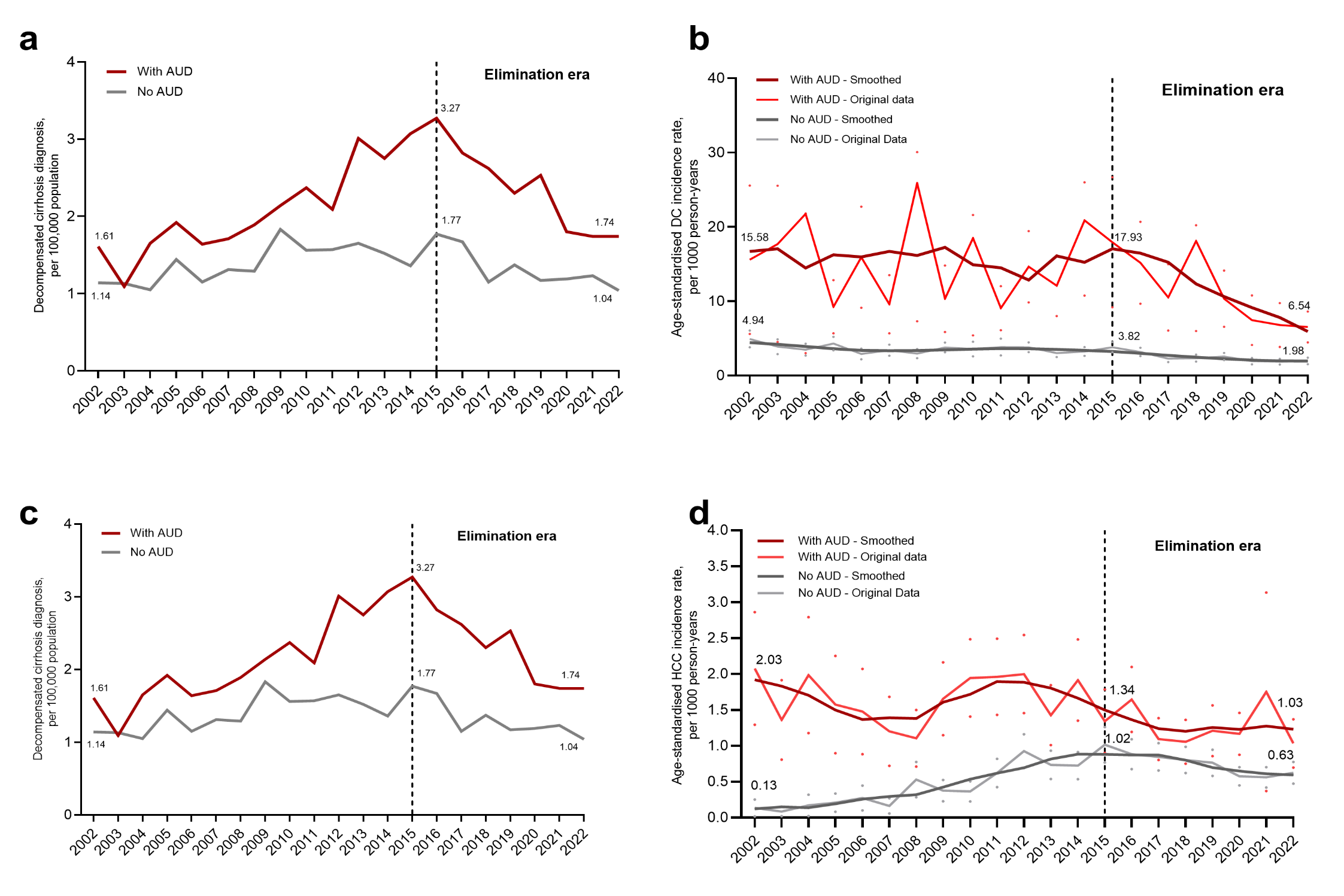


Temporal trends in (a) decompensated cirrhosis and (b) age-standardised DC incidence rates, (c) hepatocellular carcinoma and (d) age-standardised HCC incidence rates among people with an HCV notification, by alcohol-use disorder. Data from people in New South Wales, by alcohol-use disorder, 1995–2022 (n = 112,277). Age-standardised decompensated cirrhosis and hepatocellular carcinoma incidence rates were calculated per 1000 person-years and corresponding 95% CIs were calculated assuming a Poisson distribution. The Australian Standard Population 2013 was used for standardisation. DC, decompensated cirrhosis; HCC, hepatocellular carcinoma; HCV, hepatitis C virus.
